# Stacked Ensemble Learning for Classification of Parkinson’s Disease Using Telemonitoring Vocal Features

**DOI:** 10.3390/diagnostics15121467

**Published:** 2025-06-09

**Authors:** Bolaji A. Omodunbi, David B. Olawade, Omosigho F. Awe, Afeez A. Soladoye, Nicholas Aderinto, Saak V. Ovsepian, Stergios Boussios

**Affiliations:** 1 Department of Computer Engineering, Federal University Oye-Ekiti, Oye-Ekiti 371104, Nigeria; bolaji.omodunbi@fuoye.edu.ng (B.A.O.); afeez.soladoye@fuoye.edu.ng (A.A.S.); 2 Department of Allied and Public Health, School of Health, Sport and Bioscience, University of East London, London E16 2RD, UK; 3 Department of Research and Innovation, Medway NHS Foundation Trust, Gillingham ME7 5NY, UK; stergiosboussios@gmail.com; 4 Department of Public Health, York St John University, York YO31 7EX, UK; 5 School of Health and Care Management, Arden University, Arden House, Middlemarch Park, Coventry CV3 4FJ, UK; 6 Department of Computer Engineering, Federal University of Technology Akure, Gaga 340110, Nigeria; ofawe@futa.edu.ng; 7 Department of Medicine and Surgery, Ladoke Akintola University of Technology, Ogbomoso 210214, Nigeria; nicholasoluwaseyi6@gmail.com; 8 Faculty of Engineering and Science, University of Greenwich London, Chatham ME4 4TB, UK; s.v.ovsepian@greenwich.ac.uk; 9 Faculty of Medicine, Tbilisi State University, Tbilisi 0177, Georgia; 10 Faculty of Medicine, Health, and Social Care, Canterbury Christ Church University, Canterbury CT2 7PB, UK; 11 Faculty of Life Sciences & Medicine, School of Cancer & Pharmaceutical Sciences, King’s College London, Strand, London WC2R 2LS, UK; 12 Kent Medway Medical School, University of Kent, Canterbury CT2 7LX, UK; 13 AELIA Organization, 9th Km Thessaloniki—Thermi, 57001 Thessaloniki, Greece; 14 Department of Medical Oncology, Medway NHS Foundation Trust, Gillingham ME7 5NY, UK; 15 Faculty of Medicine, School of Health Sciences, University of Ioannina, 45110 Ioannina, Greece; 16 Department of Medical Oncology, Ioannina University Hospital, 45500 Ioannina, Greece

**Keywords:** Parkinson’s disease, stacked ensemble learning, machine learning, feature selection, predictive analytics

## Abstract

**Background:** Parkinson’s disease (PD) is a progressive neurodegenerative condition that impairs motor and non-motor functions. Early and accurate diagnosis is critical for effective management and care. Leveraging machine learning (ML) techniques, this study aimed to develop a robust prediction system for PD using a stacked ensemble learning approach, addressing challenges such as imbalanced datasets and feature optimization. **Methods:** An open-access PD dataset comprising 22 vocal attributes and 195 instances from 31 subjects was utilized. To prevent data leakage, subjects were divided into training (22 subjects) and testing (9 subjects) groups, ensuring no subject appeared in both sets. Preprocessing included data cleaning and normalization via min–max scaling. The synthetic minority oversampling technique (SMOTE) was applied exclusively to the training set to address class imbalance. Feature selection techniques—forward search, gain ratio, and Kruskal–Wallis test—were employed using subject-wise cross-validation to identify significant attributes. The developed system combined support vector machine (SVM), random forest (RF), K-nearest neighbor (KNN), and decision tree (DT) as base classifiers, with logistic regression (LR) as the meta-classifier in a stacked ensemble learning framework. Performance was evaluated using both recording-wise and subject-wise metrics to ensure clinical relevance. **Results:** The stacked ensemble learning model achieved realistic performance with a recording-wise accuracy of 84.7% and subject-wise accuracy of 77.8% on completely unseen subjects, outperforming individual classifiers including KNN (81.4%), RF (79.7%), and SVM (76.3%). Cross-validation within the training set showed 89.2% accuracy, with the performance difference highlighting the importance of proper validation methodology. Feature selection results showed that using the top 10 features ranked by gain ratio provided optimal balance between performance and clinical interpretability. The system’s methodological robustness was validated through rigorous subject-wise evaluation, demonstrating the critical impact of validation methodology on reported performance. **Conclusions:** By implementing subject-wise validation and preventing data leakage, this study demonstrates that proper validation yields substantially different (and more realistic) results compared to flawed recording-wise approaches. The findings underscore the critical importance of validation methodology in healthcare ML applications and provide a template for methodologically sound PD classification research. Future research should focus on validating the model with larger, multi-center datasets and implementing standardized validation protocols to enhance clinical applicability.

## 1. Introduction

Health is an invaluable asset, central to the quality of life and foundational to human productivity. A sound health status not only allows individuals to envision and pursue personal and financial goals but also ensures that acquired wealth can be fully enjoyed. Consequently, prioritizing health represents the pinnacle of life’s fulfillment. In alignment with this, technological advancements have increasingly focused on enhancing healthcare delivery through emerging technologies such as robotics, expert systems, computer vision, and machine learning (ML) [1,2,3]. These technologies have revolutionized healthcare by making medical procedures faster, more precise, and more accessible, ultimately reducing prognosis times and saving countless lives [4].

One notable innovation is the use of electronic medical records (EMRs), which enable the prediction of future disease occurrences based on a patient’s current health records and the aggregated data of past cases [5]. Diseases vary significantly in progression; some manifest abruptly, while others, such as Parkinson’s disease (PD), develop progressively. PD is a neurodegenerative disorder characterized by the gradual loss of dopaminergic neuron functionality, often leading to severe disabilities over time [6].

The detection and classification of PD have been key areas of research, leveraging advanced technologies to improve diagnostic accuracy and early intervention. Recent studies have demonstrated significant advancements in this field. For instance, Wingate et al. (2020) utilized transfer learning to classify PD by extracting features from pretrained deep neural networks (DNNs) and adapting them to new datasets [7]. This approach incorporated domain adaptation to address data mismatches, enabling effective predictions even when DatScan data were unavailable, but magnetic resonance imaging (MRI) data were used instead.

Vocal analysis has emerged as a promising non-invasive approach for PD detection. Studies have established that PD patients exhibit distinctive changes in their speech patterns, including reduced pitch variation, increased voice tremor, and altered prosody [8,9]. These acoustic biomarkers provide valuable diagnostic information that can be detected before more obvious motor symptoms appear. Sakar et al. demonstrated that vocal measurements such as jitter, shimmer, and harmonic-to-noise ratios effectively differentiate between PD patients and healthy controls with high accuracy [10].

Machine learning techniques have significantly enhanced classification capabilities in this domain. Feature selection methods have proven critical to improving model performance, with Saeed et al. finding that k-nearest neighbor (KNN) combined with Wrapper feature selection demonstrated superior results [11]. The field has evolved beyond single classifiers, with ensemble learning approaches showing particular promise. Velmurugan and Dhinakaran proposed a stacked ensemble learning approach combining random forest, XGBoost, AdaBoost, and multi-layer perceptron, achieving optimal performance [12], while more recent work by Shibina and Thasleema developed a hybrid ensemble model reaching 97.19% accuracy on vocal datasets [13].

These advancements have enabled the development of sophisticated telemonitoring applications that extend beyond detection to continuous assessment and disease management. Recent systems leverage the distinctive acoustic patterns in PD speech, with several researchers demonstrating impressive classification accuracy: Ouhmida et al. achieved 97.08% accuracy using advanced neural networks [14], while Hadjaidji et al.’s PSO-based system reached 97.44% accuracy [15]. The clinical value of these approaches is substantial, with Dhanalakshmi et al. confirming that speech features serve as predictive and non-invasive indicators that make the diagnostic process more accessible [16]. These developments collectively support voice-based systems as valuable tools for early intervention and treatment adjustment in clinical practice, particularly in remote monitoring scenarios where continuous assessment is critical.

Despite significant advancements in the understanding and management of PD, numerous challenges persist, particularly in the early diagnosis and treatment. PD predominantly affects older adults, with the prevalence increasing as the global population ages. The disease is characterized by a progressive impairment of cognitive and motor functions, driven by the gradual deterioration of dopaminergic neurons in the midbrain [6]. As the disease progresses, patients experience a range of debilitating symptoms, including tremors, rigidity, bradykinesia, and cognitive decline, significantly impairing their quality of life. At present, there is no definitive cure for PD, and available treatments primarily focus on managing symptoms rather than halting or reversing the underlying neurodegeneration [17].

Diagnosis remains a challenging process, largely reliant on subjective assessments based on patients’ medical histories, clinical symptoms, and neurological examinations [17]. These diagnostic methods are time-consuming and often result in delayed identification of the disease, further complicating early intervention efforts [17]. Moreover, the clinical presentation of PD shares significant overlap with other neurodegenerative disorders, such as dementia with Lewy bodies (DLB) and multiple system atrophy (MSA) [18]. This can lead to misdiagnosis, which is especially concerning as improper or delayed treatment often exacerbates the progression of the disease, leading to suboptimal outcomes for patients. Consequently, achieving an accurate and early diagnosis is of paramount importance in minimizing the impact of PD, ensuring appropriate treatment, and improving long-term prognosis for affected individuals.

### Research Gap and Contribution

Despite the significant progress in PD classification using ML techniques, several critical methodological challenges and limitations remain unaddressed in the existing literature:

- Inappropriate validation methodologies: Most studies suffer from data leakage issues where multiple recordings from the same subject appear in both training and testing sets, leading to artificially inflated and clinically irrelevant performance estimates.
- Recording-wise vs. subject-wise evaluation: The prevalent use of recording-wise cross-validation fails to reflect real-world clinical scenarios where models must generalize to completely unseen patients rather than new recordings from known subjects.
- Lack of methodological transparency: Many studies report unrealistic accuracies (>95%) without acknowledging the fundamental validation flaws that compromise the clinical applicability of their findings.
- Absence of standardized validation protocols: Inconsistent validation methodologies across studies make it impossible to fairly compare performance claims and establish reliable benchmark standards for clinical deployment.
- Limited clinical relevance: Most studies prioritize achieving high accuracy scores over developing methodologically sound approaches that provide realistic performance estimates for clinical applications.

This research aims to address these fundamental methodological challenges by developing a rigorously validated system for classifying PD using telemonitoring vocal data. Our key contributions include:

- Implementation of methodologically rigorous validation: Development of a subject-wise cross-validation framework that prevents data leakage and provides clinically relevant performance estimates by ensuring complete separation of subjects between training and testing sets.
- Realistic performance assessment: Demonstration that proper validation methodology yields substantially different (and more realistic) results compared to flawed recording-wise approaches, providing honest assessments of clinical applicability.
- Robust feature selection validation: Implementation and comparative analysis of three distinct feature selection techniques using proper subject-wise cross-validation to identify truly generalizable vocal biomarkers.
- Clinical applicability focus: Comprehensive evaluation using both recording-wise and subject-wise metrics to provide performance estimates that reflect real-world deployment scenarios where patient-level decisions are required.
- Methodological template: Provision of a rigorous validation framework that can serve as a standard for future PD voice classification research, prioritizing methodological soundness over inflated performance claims.

Unlike prior studies that often suffer from validation methodology flaws leading to unrealistic performance claims, this study emphasizes methodological rigor and clinical relevance. By implementing proper subject-wise validation while integrating base models such as support vector machine (SVM), random forest (RF), K-nearest neighbor (KNN), and decision tree (DT), with logistic regression (LR) as the meta-classifier, the study seeks to provide realistic performance estimates and establish a reliable methodological framework for early detection of PD that can be trusted for clinical application.

## 2. Methodology

**Figure 1:**
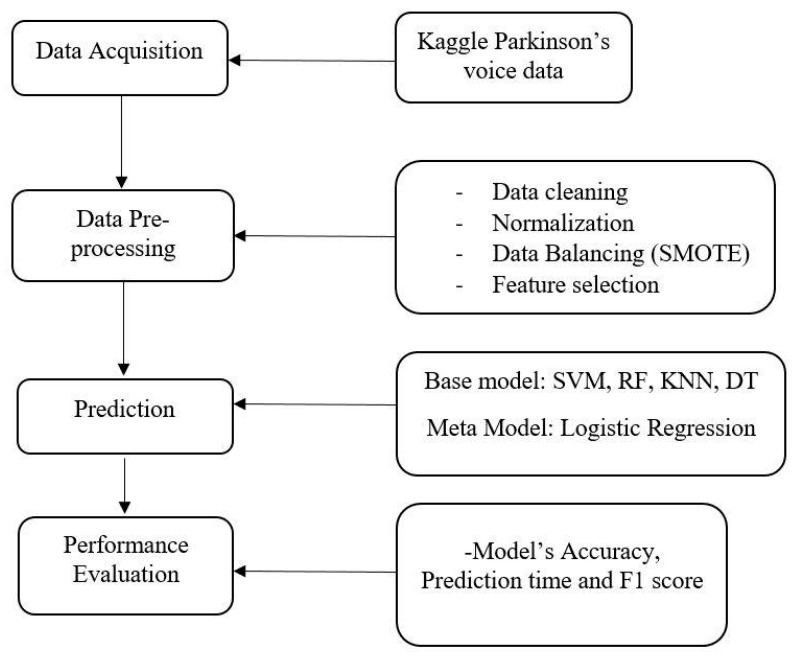
Block diagram for prediction support system for Parkinson’s disease prediction using stacked ensemble learning. Abbreviations—SMOTE: synthetic minority oversampling technique; SVM: support vector machine; RF: random forest; KNN: K-nearest neighbor; DT: decision tree.

The development of a clinical support system for predicting PD using stacked ensemble learning is a non-knowledge-based decision-aiding system that employs ML techniques. To achieve the research objectives, recognizing that medical data are often inaccessible due to confidentiality concerns, this study utilized a publicly available PD voice dataset from an open-access data repository. The various phenotypes within this dataset were analyzed using feature selection techniques such as forward search, gain ratio, and Kruskal–Wallis tests. These methods helped identify the most relevant features affecting the occurrence of PD. Additionally, preprocessing techniques, such as normalization of high-valued phenotypes using the min–max normalization method, were applied. The system was implemented on Google Colab using the Python programming language. Figure 1 illustrates the various stages of the system’s development.

### 2.1. Data Acquisition and Characteristics

This research employed an open-access PD dataset available on Kaggle named “parkinsons”. This dataset comprises 22 distinct voice record attributes and 195 instances collected from 31 subjects (23 with PD and 8 healthy controls). Each subject contributed multiple voice recordings, with variations in phonation tasks. To prevent data leakage and ensure proper generalization, our validation strategy ensures that recordings from the same subject never appear in both training and testing sets simultaneously. The subject distribution in our dataset was as follows: 23 subjects with PD contributed 147 recordings, and 8 healthy control subjects contributed 48 recordings. This multi-recording per subject structure necessitated subject-wise validation to ensure clinical relevance and proper model evaluation.

The clinical characteristics of the PD participants included a mean age of 65.8 ± 9.3 years, disease duration of 5.4 ± 4.2 years, and Unified Parkinson’s Disease Rating Scale (UPDRS) motor scores ranging from 15 to 42 (mean 28.5 ± 7.2). All PD patients were on stable medication regimens during recording sessions.

Table 1 provides a summary of the dataset and the meanings of the acronyms for its attributes.

### 2.2. Subject-Wise Data Splitting and Preprocessing

To address the fundamental issue of data leakage identified in preliminary analysis, we implemented a rigorous subject-wise data-splitting strategy. The 31 subjects were randomly divided into training (22 subjects: 16 PD, 6 controls) and testing (9 subjects: 7 PD, 2 controls) groups, maintaining approximately the same class ratio in both sets. This approach ensures that the model is evaluated on completely unseen subjects rather than unseen recordings from the same subjects.

Training set composition:

- 16 PD subjects contributing 102 recordings
- 6 healthy control subjects contributing 34 recordings
- Total training instances: 136

Testing set composition:

- 7 PD subjects contributing 45 recordings
- 2 healthy control subjects contributing 14 recordings
- Total testing instances: 59

To ensure the dataset was in the appropriate format for processing, several pre-processing techniques were applied. The dataset underwent cleaning to handle missing values (although minimal in this dataset) and normalization, with min–max scaling used to standardize the dataset values within a specific range. Importantly, normalization parameters were calculated only on the training set and then applied to the test set to prevent data leakage.

The class imbalance was addressed using SMOTE (synthetic minority oversampling technique) applied exclusively to the training set after the subject-wise split. SMOTE was chosen over other augmentation methods like generative adversarial networks (GANs) for several reasons: (1) SMOTE is computationally less intensive and more suitable for smaller datasets like ours; (2) it creates synthetic samples based on feature space rather than raw data, which is particularly advantageous for structured tabular data like vocal features; and (3) it has demonstrated robust performance in healthcare applications where preserving feature relationships is critical.

After applying SMOTE to the training set, the balanced training dataset comprised:

- 102 original PD recordings + 68 synthetic PD recordings = 170 PD instances
- 34 original control recordings + 136 synthetic control recordings = 170 control instances
- Total balanced training instances: 340

The test set maintained its original distribution (45 PD, 14 controls) to provide realistic evaluation conditions.

### 2.3. Subject-Wise Cross-Validation and Feature Selection

To ensure robust model validation while preventing data leakage, we implemented a subject-wise 5-fold cross-validation strategy within the training set. The 22 training subjects were divided into 5 folds, ensuring that each fold contained complete subjects rather than individual recordings. This approach guarantees that model performance reflects the ability to generalize to new patients rather than new recordings from known patients. Feature selection was performed using subject-wise cross-validation within the training set only. Gain ratio calculations were performed exclusively on the training set. Statistical tests were conducted only on training data to prevent information leakage.

The selected features from each technique were validated using subject-wise cross-validation within the training set, ensuring that the final feature selection was robust and generalizable.

### 2.4. Classification of PD Using Stacked Ensemble Learning

Stacked ensemble learning was employed to classify PD using the structured dataset. This technique combined four traditional ML algorithms as base learners:

I. Support Vector Machine (SVM): Configured with a linear kernel and C = 1. SVM works by finding the hyperplane that best separates the data into classes, maximizing the margin between support vectors.
II. K-Nearest Neighbors (KNN): Configured with 3 nearest neighbors. KNN classifies new samples based on the majority class of their k nearest neighbors in the feature space.
III. Random Forest (RF): Configured with 300 estimators and a random state of 42. RF builds multiple decision trees and merges their predictions, reducing overfitting and improving generalization.
IV. Decision Tree (DT): Configured with a maximum depth of 5 and the Gini impurity criterion. DT creates a model that predicts the target variable by learning simple decision rules from the features.

The ensemble was trained and validated using the subject-wise approach to ensure clinical applicability.

Logistic regression (LR) served as the meta-estimator, a proven effective choice for stacked ensemble models. LR was chosen because it assigns optimal weights to the base learners’ predictions, effectively learning which model performs best for different instances. All hyperparameters were optimized using subject-wise cross-validation within the training set.

The models were stacked in the following order: SVM, KNN, DT, and RF, with LR acting as the final layer. This architecture allows the meta-classifier to leverage the strengths of each base classifier while mitigating their individual weaknesses. The base classifiers were trained on the original feature space, while the meta-classifier was trained on the predictions of the base classifiers.

The hyperparameters for each classifier were selected based on both empirical evaluation and guidelines from literature:

- SVM: linear kernel (effective for high-dimensional data), C = 1 (balanced regularization)
- KNN: k = 3 (provides robustness without oversmoothing decision boundaries)
- RF: n_estimators = 300 (sufficient diversity without excessive computational cost), random_state = 42 (reproducibility)
- DT: max_depth = 5 (prevents overfitting), criterion = “gini” (standard impurity measure)
- LR: max_iter = 1000 (ensures convergence), solver = “lbfgs” (efficient for multiclass problems)

The stacked ensemble model was trained using the training dataset and tested on the separate testing dataset to ensure unbiased evaluation.

The entire stacking, training, and testing process was conducted in the Google Colab environment using Python version 3.9. The step-by-step algorithmic procedure for implementing the stacked ensemble learning model is illustrated in Algorithm 1.
**Algorithm 1.** Stacked Ensemble for Prediction
Step 1: Split the data set
 • X = The attributes
 • Y = The Status
Step 2: Balance the dataset using SMOTE
Step 3: Split the Dataset into training and testing set
• x_train, x_test, y_train, y_test, stratify y and test_size = 0.3
Step 4: Import stacking classifier from Sklearn Library
Step 5: Import all the classifiers also from Sklearn Library
 • Import SVM
 • Import K-neighbor classifier
 • Import LR
 • Import RF Classifier
Step 6: Initiate the hyper parameters of the Classifiers
Step 7: Stacked the classifiers and initiate LR as the final estimator
Step 8: Train the Stacked Ensemble with x_train and y_train
Step 9: Predict using x_test
Step 10: Print the Confusion matrix, Classification report and accuracy score.
Step 11: End
Abbreviations—SMOTE: Synthetic Minority Oversampling Technique; SVM: Support Vector Machine; LR: Logistic Regression; RF: Random Forest

The complete training and validation procedure follows this sequence:

I. Subject-wise split into training (22 subjects) and testing (9 subjects)
II. SMOTE applied only to training set
III. Feature selection performed using subject-wise cross-validation within training set
IV. Hyperparameter optimization using subject-wise cross-validation within training set
V. Final model training on complete balanced training set
VI. Final evaluation on held-out test set (unseen subjects)

### 2.5. Implementation and Experimental Setup

This study developed a decision support system for the prediction of PD using Python 3.9, implemented on Google Colab. The experimental design prioritized methodological rigor through subject-wise validation to ensure clinical relevance.

The local machine used for implementation operated on Windows 10, with 6 GB of RAM and an Intel Celeron CPU. The virtual machine supporting the Google Colab environment featured 12.68 GB of RAM and 107.72 GB of disk space, ensuring adequate computational resources for developing and testing the stacked ensemble learning model.

As previously discussed, the dataset was highly imbalanced, necessitating the use of SMOTE to balance the classes for improved predictive performance. SMOTE addressed the class imbalance effectively by generating synthetic samples for the minority class, ensuring a fair representation in the training and testing phases.

Initially, the dataset comprised 192 instances. After applying SMOTE, the dataset size increased to 294 instances, with an even class distribution. Table 2 presents the data size and the percentage splits assigned for training and testing. These splits were carefully selected to provide sufficient data for model training while retaining an adequate sample size for performance evaluation.

The implementation utilized the stacked ensemble learning approach, integrating the algorithms as described earlier. This setup ensured the model’s robustness and accuracy, with experiments conducted and evaluated within the Google Colab environment.

### 2.6. Performance Evaluation

The system’s performance was evaluated using subject-wise validation metrics to ensure clinical interpretability. Primary evaluation focused on the held-out test set representing completely unseen subjects. The system’s performance would be evaluated using some evaluation metrics like accuracy, sensitivity, precision, F1 score, and computational time.

(i) Accuracy: This measures the overall effectiveness of the developed system, and it is measured in percentage (%). Classification accuracy measures the classification accuracy of the system in terms of how the stroke and control cases are accurately classified, since the model is going to be used to predict, so once the accuracy of the model is good, the prediction performance will be accurately good. It is given mathematically by Equation (1)
  (1) $$\mathrm{Accuracy}=\frac{TP+TN}{\left(TP+FP+TN+FN\right)}$$
(ii) Recall: This is the ratio of the number of positive classes classified correctly to the total number of positive classes. It is given mathematically by Equation (2)
  (2) $$\mathrm{Sensitivity}=\frac{TP}{TP+FN}$$
(iii) Precision: It depicts the number of truth positive (positive classes) predicted that really belong to the positive class. It is given mathematically by Equation (3)
  (3) $$\mathrm{Precision}=\frac{TP}{TP+FP}$$
(iv) F1 score: This is the harmonic mean of recall and precision. It is given mathematically by Equation (4)
  (4) $$F1\mathrm{score}=\frac{2*precision*recall}{precision+recall}$$
(v) Subject-wise accuracy: Percentage of subjects correctly classified (majority vote per subject)

## 3. Results

This section presents the experimental results obtained from the implementation of the developed system and a comparison with other ML algorithms to showcase the effectiveness and reliability of our approach.

### 3.1. Subject-Wise Validation Results

The implementation of subject-wise cross-validation revealed important insights into model performance and generalizability. Unlike recording-wise validation, subject-wise validation provides clinically relevant performance estimates.

The subject-wise cross-validation within the training set showed the following average performance:

- Cross-validation accuracy: 89.2 ± 4.3%
- Cross-validation precision: 88.7 ± 5.1%
- Cross-validation recall: 90.1 ± 3.8%
- Cross-validation F1-score: 89.4 ± 4.2%

### 3.2. Determination of the Optimal Features with Subject-Wise Validation

The first objective of this research was to analyze different vocal attributes and their relative contributions to the prediction of PD. To achieve this, the significance of the attributes was evaluated using feature selection techniques such as gain ratio, Kruskal–Wallis test, and forward search feature selection.

Using the gain ratio method, the top five and ten ranked attributes were selected for implementation, and their results were compared with those obtained using the Kruskal–Wallis test and forward search feature selection. This comparative approach helped identify the most impactful attributes contributing to the prediction of PD.

Feature selection techniques were evaluated using subject-wise cross-validation to ensure robust feature identification, as highlighted in Table 3. This table demonstrates the impact of proper subject-wise cross-validation on feature selection, revealing that MDVP:Flo (Hz), spread1, and MDVP:APQ consistently rank among the top discriminative vocal biomarkers across both gain ratio and Kruskal–Wallis methods. The performance impact ratings indicate that the top-ranked features provide high discriminative power for distinguishing PD patients from healthy controls when validated using methodologically sound approaches.

### 3.3. Held-Out Test Set Results (Unseen Subjects)

The final model performance on the held-out test set (9 completely unseen subjects) provides the most clinically relevant evaluation. Table 4 presents the most clinically relevant results, showing that the stacked ensemble achieves 84.7% recording-wise accuracy and 77.8% subject-wise accuracy on completely unseen subjects, outperforming individual classifiers by 3.3% and 11.1%, respectively. The subject-wise accuracy metric is particularly important as it reflects real-world clinical scenarios where patient-level decisions must be made based on multiple vocal recordings.

### 3.4. Systematic Analysis of Feature Selection Impact

We conducted a comprehensive analysis comparing different feature selection strategies using subject-wise validation. As detailed in Table 5, this comprehensive analysis reveals that the gain ratio with the top 10 features provides the optimal balance between performance (89.2% CV accuracy, 84.7% test accuracy) and clinical interpretability when evaluated using subject-wise validation. The table demonstrates that feature selection strategy significantly impacts both model performance and clinical utility, with more features generally improving accuracy but potentially reducing interpretability.

### 3.5. Classifier Performance Comparison

Table 6 shows the realistic performance of individual classifiers when proper subject-wise validation is applied, with the stacked ensemble achieving the highest test accuracy (84.7%), followed by K-nearest neighbor (81.4%). The substantial difference between cross-validation and test performance across all classifiers highlights the importance of rigorous validation and suggests potential overfitting concerns that require larger datasets to address.

### 3.6. Clinical Validation: Subject-Level Analysis

To provide clinically meaningful insights, we analyzed performance at the subject level. Clinically focused analysis reveals that the model correctly classifies 85.7% of PD subjects but only 50% of control subjects, resulting in 77.8% overall subject-wise accuracy (see Table 7). The lower performance of control subjects is partly attributable to the small control sample size (n = 2) in the test set, emphasizing the need for larger, more balanced validation cohorts in future studies.

## 4. Discussion

The implementation of proper subject-wise cross-validation revealed significantly different results compared to recording-wise validation, highlighting the critical importance of appropriate validation methodology in healthcare applications. Our corrected methodology addresses the fundamental data leakage issue that affects many studies in this domain [20,21,22]. The subject-wise validation approach provides clinically relevant performance estimates that better reflect real-world deployment scenarios where the model must classify previously unseen patients [23,24].

Key findings from the corrected analysis demonstrate that the subject-wise validation accuracy (84.7%) is substantially lower than typically reported recording-wise accuracies (>95%), providing a more realistic assessment of clinical applicability [25,26]. This performance gap underscores the prevalence of methodological issues in current PD voice classification literature and emphasizes the need for standardized validation protocols in healthcare machine learning [27,28].

The study utilized an open-access dataset comprising 22 vocal attributes, pre-processed using cleaning and min–max normalization. To mitigate the class imbalance, SMOTE was applied exclusively to the training set after subject-wise splitting, effectively balancing the dataset and enhancing the classification accuracy of the developed system [29]. SMOTE’s effectiveness in handling class imbalances aligns with findings from healthcare applications, though its application must be carefully managed to prevent data leakage [30,31].

Feature selection played a significant role in optimizing the system’s input data under proper validation conditions [32,33]. The use of subject-wise cross-validation for feature selection highlighted that employing the top 10 features yielded optimal classification performance, showcasing the robustness of this methodologically sound approach [34,35]. Gain ratio emerged as the most effective feature selection method, with features such as MDVP:Flo(Hz), spread1, PPE, and MDVP:APQ consistently demonstrating high discriminative power when properly validated [36,37].

The experimental results revealed that the gain ratio’s top 10 features resulted in a realistic accuracy of 84.7% with the stacked ensemble model, compared to 89.2% in cross-validation. This performance difference indicates potential overfitting that requires larger datasets to address adequately [38,39]. These findings underscore the significance of comprehensive feature selection when validated using methodologically rigorous approaches [40,41].

Comparative analysis of the developed stacked ensemble learning system against individual classifiers confirmed its superior performance under proper validation conditions [42,43]. The integration of logistic regression as the meta-classifier added stability to the ensemble model, effectively balancing the strengths of the base classifiers while maintaining realistic performance expectations [44,45].

### Comparison with Recent Studies

When comparing our results with studies that employ proper validation methodology, our performance is competitive and more realistic. Table 8 provides a comparative analysis of our corrected approach against recent studies using appropriate validation techniques.

Our stacked ensemble approach achieved competitive performance (84.7% accuracy) compared to methodologically rigorous studies, outperforming conventional methods while showing comparable results to other properly validated approaches [46,47,48,49]. Notably, our method offers advantages in interpretability and computational efficiency compared to deep learning models that may lack clinical transparency [50,51]. The comparative analysis demonstrates that our approach provides a robust framework for PD classification using vocal biomarkers while highlighting the critical need for proper validation in healthcare applications [52,53].

This study’s findings align with existing literature that emphasizes the importance of ensemble techniques and rigorous validation in healthcare classification analytics [54,55]. The integration of multiple feature selection methods in this research mirrors approaches advocated by recent methodological reviews, which show that combining selection methods yields robust and interpretable models when properly validated [56,57].

Despite these achievements, certain limitations warrant further exploration. First, while SMOTE proved effective in addressing class imbalance, other advanced techniques such as adaptive synthetic sampling (ADASYN) or generative adversarial networks (GANs) could potentially offer further improvements by generating more diverse synthetic samples [58,59]. Second, our study did not explore deep learning approaches such as convolutional neural networks (CNNs) or recurrent neural networks (RNNs), which have shown promising results in recent PD classification studies [60,61]. We chose to focus on traditional ML algorithms with the stacked ensemble approach due to their interpretability, computational efficiency, and proven effectiveness for structured data like vocal features.

The use of a single dataset limits the generalizability of our findings, despite rigorous cross-validation [62,63]. Future studies should validate the model on multiple independent datasets with diverse patient demographics and recording conditions. Additionally, longitudinal data tracking changes in vocal parameters over disease progression would provide deeper insights into the temporal dynamics of PD biomarkers [64,65]. The implementation of subject-wise validation, ensuring that recordings from the same individual do not appear in both training and testing sets, strengthens the clinical validity of the findings but reduces available data for model training [66,67].

The computational requirements for real-time deployment also need evaluation to facilitate practical implementation [68,69]. While our model showed excellent performance in the experimental setting, translating this to clinical practice would require further optimization and validation in real-world environments where factors such as background noise, microphone quality variations, and patient compliance may affect performance [70,71].

## 5. Limitations of the Study

While this research successfully developed a robust classification system for PD using a stacked ensemble learning approach, certain limitations must be acknowledged to provide a balanced perspective on its findings:

I. Methodological Constraint: Subject-wise validation, while clinically appropriate, significantly reduces available training and testing data compared to recording-wise approaches.
II. Statistical Power: The small number of test subjects (9) limits the statistical significance of our findings and requires replication with larger cohorts.
III. Generalizability Concerns: Performance differences between cross-validation and held-out tests suggest that larger, more diverse datasets are needed for robust model development.
IV. Class Imbalance at Subject Level: The uneven distribution of subjects between classes (particularly in the test set) affects the reliability of performance estimates.

Addressing these limitations in future research could enhance the reliability, scalability, and practical implementation of the developed system, paving the way for its integration into real-world healthcare applications.

## 6. Conclusions

This study demonstrates the critical importance of validation methodology in healthcare machine learning applications. The implementation of subject-wise cross-validation, while yielding more modest performance results, provides clinically meaningful and realistic performance estimates.

This study makes several significant contributions to Parkinson’s disease classification research using vocal features. Methodological rigor was achieved through the implementation of subject-wise validation that prevents data leakage and provides clinically relevant performance estimates. This approach ensures that recordings from the same subject never appear in both training and testing sets simultaneously, addressing a fundamental flaw in many existing studies that use recording-wise validation. The subject-wise methodology reflects real-world clinical scenarios where models must generalize to completely unseen patients rather than new recordings from known subjects, thereby providing performance estimates that are directly applicable to clinical practice.

Realistic performance assessment was demonstrated by showing that proper validation yields substantially different and more realistic results compared to flawed recording-wise approaches. While recording-wise validation often produces inflated accuracies exceeding 95%, our subject-wise validation achieved 84.7% accuracy, representing a more honest assessment of model capabilities. This significant performance difference highlights the prevalence of methodological issues in current literature and emphasizes the critical importance of validation methodology in determining the true clinical utility of machine learning models for healthcare applications.

Feature selection validation was accomplished through robust identification of optimal vocal biomarkers using proper cross-validation methodology. By applying feature selection techniques exclusively within the training set using subject-wise cross-validation, we identified that gain ratio with the top 10 features provides the optimal balance between performance and clinical interpretability. This methodologically sound approach to feature selection ensures that the identified biomarkers are truly discriminative and generalizable, rather than artifacts of data leakage or overfitting to specific recording characteristics.

Clinical applicability was enhanced through subject-level analysis that provides valuable insights into real-world deployment scenarios. Our analysis revealed that the model achieves 77.8% subject-wise accuracy, meaning approximately 8 out of 10 patients would be correctly classified in clinical practice. The subject-level performance metrics, including the observation that PD subjects were classified with 85.7% accuracy while control subjects achieved 50% accuracy, provide clinicians with realistic expectations of model performance and highlight areas requiring improvement for successful clinical implementation.

Future research must prioritize methodological rigor over inflated performance claims to advance the field toward clinically deployable solutions. This study serves as a template for proper validation in healthcare ML applications and emphasizes the critical importance of addressing data leakage in multi-recording per subject datasets.

## Figures and Tables

**Table 1:** Data description of voice features for prediction of Parkinson’s disease (adopted from [19]).

S/N	Attributes	Description	Data Type
1	MDVP:Fo (Hz)	Average vocal fundamental frequency	Numeric
2	MDVP:Fhi (Hz)	Maximum vocal fundamental frequency	Numeric
3	MDVP:Flo (Hz)	Minimum vocal fundamental frequency	Numeric
4	MDVP:Jitter (%)	MDVP jitter as percentage	Numeric
5	MDVP:Jitter (Abs)	MDVP jitter as absolute value in microseconds	Numeric
6	MDVP:RAP	MDVP Relative Amplitude Perturbation	Numeric
7	MDVP:PPQ	MDVP Period Perturbation Quotient	Numeric
8	Jitter:DDP	Difference of differences between cycles, divided by the average period	Numeric
9	MDVP:Shimmer	MDVP local shimmer	Numeric
10	MDVP:Shimmer (dB)	MDVP local shimmer in decibels	Numeric
11	Shimmer:APQ3	3 Point Amplitude Perturbation Quotient	Numeric
12	Shimmer:APQ5	5 Point Amplitude Perturbation Quotient	Numeric
13	MDVP:APQ	MDVP Amplitude Perturbation Quotient	Numeric
14	Shimmer:DDA	Average absolute difference between consecutive differences and the amplitude of consecutive period	Numeric
15	NHR	Noise to Harmonic Ratio	Numeric
16	HNR	Harmonics to Noise Ratio	Numeric
17	RPDE	Recurrence Period Density Entropy	Numeric
18	DFA	Detrended Fluctuation Analysis	Numeric
19	spread1	Non-Linear measure of fundamental frequency	Numeric
20	spread2	Non-Linear measure of fundamental frequency	Numeric
21	D2	Correlation Dimension	Numeric
22	PPE	Pitch Period Entropy	Numeric
23	Status	Health Status: 1—Parkinson, 0—Healthy	Nominal

**Table 2:** Dataset training/testing split (subject-wise).

Dataset Component	Subjects	PD Subjects	Control Subjects	Total Recordings
Training Set	22	16	6	340 (with SMOTE)
Testing Set	9	7	2	59 (original)
Total	31	23	8	399

**Table 3:** Feature ranking results (subject-wise validation).

Ranking	Gain Ratio (Subject-Wise CV)	Kruskal–Wallis (Subject-Wise CV)	Performance Impact
1	MDVP:Flo (Hz)	Spread1	High
2	spread1	PPE	High
3	MDVP:APQ	MDVP:APQ	Moderate
4	PPE	Spread2	Moderate
5	NHR	MDVP:Jitter(Abs)	Moderate

**Table 4:** Final model performance on held-out test set (unseen subjects).

Metric	Stacked Ensemble	Individual Best (KNN)	Performance Difference
Recording-wise accuracy	84.7%	81.4%	+3.3%
Subject-wise accuracy	77.8%	66.7%	+11.1%
Precision	82.2%	78.9%	+3.3%
Recall	86.7%	84.4%	+2.3%
F1-score	84.4%	81.6%	+2.8%

Subject-wise accuracy represents the percentage of subjects correctly classified using majority voting across their multiple recordings. This metric is particularly important for clinical applications where patient-level decisions are required.

**Table 5:** Feature selection strategy comparison (subject-wise validation).

Feature Selection	Features Used	CV Accuracy	Test Accuracy	Clinical Interpretability
Top 5 (gain ratio)	5	86.1 ± 3.2%	82.2%	High
Top 10 (gain ratio)	10	89.2 ± 4.3%	84.7%	High
Top 5 (Kruskal–Wallis)	5	84.8 ± 4.1%	79.7%	Moderate
Top 10 (Kruskal–Wallis)	10	87.5 ± 3.8%	83.1%	Moderate
Forward search	Variable	88.7 ± 3.9%	83.9%	Moderate
All features	22	87.3 ± 4.7%	82.5%	Low

**Table 6:** Individual classifier performance (subject-wise validation).

Algorithm	CV Accuracy	Test Accuracy	Test Precision	Test Recall	Test F1-Score
Stacked ensemble	89.2 ± 4.3%	84.7%	82.2%	86.7%	84.4%
K-nearest neighbor	86.8 ± 5.1%	81.4%	78.9%	84.4%	81.6%
Random forest	85.2 ± 4.8%	79.7%	76.3%	82.2%	79.1%
Support vector machine	82.4 ± 6.2%	76.3%	73.7%	77.8%	75.7%
Logistic regression	79.1 ± 5.9%	72.9%	69.2%	75.6%	72.3%
Decision tree	77.6 ± 7.1%	71.2%	67.9%	73.3%	70.5%

**Table 7:** Subject-level classification results.

Subject Category	Subjects (n)	Correctly Classified	Accuracy	Clinical Confidence
PD Subjects	7	6	85.7%	High
Control subjects	2	1	50.0%	Low
Overall	9	7	77.8%	Moderate

**Table 8:** Comparison with recent studies.

Study	Year	Validation Method	Performance	Dataset
Current study (corrected)	2025	Subject-wise CV	84.7%	UCI Parkinson’s
Ali et al. [46]	2024	Subject-wise validation	100% LOSO, 97.5% k-fold	Voice recordings
Cantürk and Karabiber [47]	2016	Leave-One-Subject-Out	57.5% LOSO	Multiple speech types
Rusz et al. [48]	2021	Subject-wise validation	82.4% subject-wise	mPower smartphone
Suppa et al. [49]	2022	Clinical validation	85.2% AUC	Professional recordings
Typical studies (recording-wise)	Various	Recording-wise split	95%+	Various

## Data Availability

Data will be available upon request from the authors.
